# Perioperative nutritional interventions for gastric cancer patients: a meta-analysis of surgical outcomes

**DOI:** 10.3389/fnut.2025.1680833

**Published:** 2025-10-29

**Authors:** QiJun Li, Chen Chen, XiaoHong Wang

**Affiliations:** General Surgery (Gastroenterology), Huadong Hospital, Fudan University, Shanghai, China

**Keywords:** gastric cancer, nutritional interventions, immunonutrition, oral nutritional supplements, dietary counseling, multimodal nutrition, randomized controlled trials, meta-analysis

## Abstract

**Objective:**

To assess the effectiveness of nutritional interventions such as multimodal nutrition interventions, dietary education and digital interventions, immunonutrition, and oral nutritional supplements (ONS) on clinical, immunological, and biochemical outcomes among gastric cancer patients.

**Background:**

Nutritional interventions become a key element in enhancing clinical, immunological, and biochemical outcomes in those who have gastric cancer and are receiving surgery, chemotherapy, or follow-up care after discharge.

**Methods:**

Eighteen randomized controlled trials (RCTs) were included, examining interventions like multimodal nutrition (EN, PN, probiotics, early oral feeding), dietary counseling, digital platforms, immunonutrition (*ω*-3, arginine, RNA-enriched formulas), and ONS. Primary outcomes were albumin (Nutritional marker used to assess malnutrition), CRP(C-reactive protein biomarker for systemic inflammation), IgA, interleukins, weight, quality of life, complications, and mortality. Data were pooled with a random-effects model with inverse variance methods to estimate standardized mean differences (SMD) with 95% confidence intervals (CI). Heterogeneity was measured using the *I*^2^ statistic.

**Results:**

Separate studies indicated the following benefits with respective interventions: improvement in albumin, CRP, immunological markers, weight, quality of life, and fewer complications. Pooled analyses, however, revealed no statistically significant effects overall: multimodal nutrition (SMD −0.43, 95% CI −0.99 to 0.13), dietary counseling/education/digital interventions (SMD 0.27, 95% CI −0.34 to 0.88), immunonutrition (SMD −0.16, 95% CI −0.58 to 0.26), and ONS (SMD 0.07, 95% CI −0.27 to 0.41). Strong heterogeneity was identified for all interventions (*I*^2^ range: 85–88%, *p* < 0.01), indicating variability in study populations, intervention types, durations, and outcomes measured.

**Conclusion:**

Although single trials indicate nutritional interventions can enhance biochemical, immunological, and clinical responses in gastric cancer patients, pooled evidence fails to show overall consistent benefits. Substantial heterogeneity emphasizes the need for larger, uniform trials to establish proper nutritional interventions, formulations, and timing. These findings validate the potential usefulness of individually tailored nutritional support as an adjunct to conventional treatment.

## 1 Introduction

The most prevalent and lethal type of cancer worldwide is gastric cancer, especially in East Asia, with frequency much higher than that of Western populations. Even with the progress in surgery, chemotherapy, and targeted therapy, Patients with stomach cancer continue to have a low survival rate and a high death rate. One of the most potent issues in gastric cancer management is the risk of malnutrition, already existing at presentation and further augmented by aggressive therapeutic approaches (1). Malnutrition among gastric cancer patients correlates with reduced tolerance to treatment, higher rates of postoperative complications, longer hospital stays, and worse overall survival. For these concerns, perioperative nutrition therapy evolved into an area of concern over a possibly preventable intervention supporting the prognosis in gastric cancer surgery (2).

The perioperative phase, spanning from perioperative, intraoperative, to postoperative times, represents an influential period of opportunity for the use of nutritional therapy. Supportive feeding throughout this window of time has proven to reinforce immunity, aid wound healing, diminish infection incidence, and, overall, promote the survival of the patient. There have been several approaches to perioperative nutrition utilized, some being enteral nutrition (EN), parenteral nutrition (PN), immunonutrition, and individualized dietetic modulations on an as-needed basis. Gastric cancer patients experience anorexia, dysphagia, and secondary tumor burden-associated metabolic derangement with consequent relevant weight loss. Early identification of high-risk patients can be addressed by comprehensive nutritional risk screening with tools such as the Nutritional Risk Screening 2002 (NRS-2002) and the Patient-Generated Subjective Global Assessment (PG-SGA). Evidence indicates that perioperative immune nutrient supplementation with arginine, *ω*-3 fatty acids, and glutamine can improve immune function (the ability of the body to fight against the pathogens) and decrease postoperative complications. Perioperative carbohydrate loading has also been hailed as an element of enhanced recovery after surgery (ERAS) protocols for optimizing insulin sensitivity and minimizing postoperative catabolism (3).

During intraoperative periods, it is still challenging to provide adequate nutrition because patients have prolonged periods of fasting. Perioperative fasting used to be encouraged to avoid aspiration during anesthesia, but present guidelines suggest a more liberal practice with a focus on the need to provide metabolic homeostasis. Intravenous glucose infusion and early enteral nutrition following surgery can be utilized to ensure energy balance as well as avoid excessive muscle catabolism. In addition, newer studies have shown the advantages of perioperative immunonutrition, including supplementation with certain bioactive molecules involved in immune and inflammatory modulation. Immunonutrition-rich diets enhanced with *ω*-3 fatty acids, nucleotides, and arginine have been associated with favorable surgical results, fewer infectious complications, and shorter hospital stays among patients with gastric cancer. Postoperative nutrition is equally as important in providing an uneventful recovery. Gastrectomy, especially total or subtotal gastrectomy, functionally and anatomically changes the gastrointestinal tract and lays the groundwork for complications of delayed gastric emptying, dumping syndrome, and malabsorption. Early enteral feeding, where possible, is favored over parenteral nutrition because it has the potential to maintain intestinal integrity and facilitate earlier recovery (4). But in patients with intolerance, complete parenteral nutrition (TPN) might be required to support caloric and protein needs. According to recent meta-analyses, patients who get early enteral or oral nutrition following gastric cancer resection had higher overall functional status, fewer infection problems, and shorter hospital stays. Additionally, to address deficits resulting from gastric resection, micronutrient supplements in the form of vitamins B12, D, and iron are typically required (4).

Notwithstanding the accumulation of evidence to support perioperative nutritional therapy, some lacunae are still in the literature. Optimal composition, timing, and duration of nutrition support strategies continue to be controversial, with no consensus on putting standardized protocols into practice. Also, heterogeneity among individuals for nutritional status, tumor burden, and metabolic response complicates creating a one-size-fits-all solution. All the prior studies have limited sample sizes, patient selection bias, and methodological flaws that preclude generalizability of findings. Systematic review and meta-analysis are hence necessary to synthesize existing evidence, determine the most effective nutritional interventions, and make clear clinical recommendations for optimal perioperative nutrition in gastric cancer patients (3). A number of randomized controlled trials (RCTs) have compared the effectiveness of such nutritional interventions among gastric cancer patients about to undergo surgery, but results are still inconsistent within individual trials, with differences in intervention types, dose, timing, and outcomes measured (5). Although previous systematic reviews have investigated nutritional interventions among more general gastrointestinal or oncologic patient groups, a dedicated meta-analysis combining solely RCT evidence among gastric cancer patients in the perioperative setting is rare (6). In order to fill this gap, the current study systematically searches and quantitatively analyses RCTs assessing the efficacy of perioperative nutrition treatment on surgical outcomes in gastric cancer patients. Outcomes of interest include postoperative nutritional status, inflammatory markers, immune function, complications, and short-term recovery indicators. Through stratification by intervention type, this review aims to provide more precise and clinically relevant guidance regarding nutritional management for this at-risk patient group. This meta-analysis was aimed at establishing the efficacy of several perioperative nutritional therapy regimens on patients with gastric cancer undergoing surgery. The primary objectives include ascertaining the impact of treatment on post-operative complications, in-patient hospital stay, tolerability of therapy, immune function, and mortality. By synthesizing evidence from randomized controlled trials (RCTs), the present review aims to provide evidence-based recommendations that can be practical in clinical practice and help enhance patient outcomes. The present study will also examine subgroup differences according to patient characteristics, and modalities of nutritional intervention to enhance personalized nutritional regimens for gastric cancer treatment.

## 2 Methods

### 2.1 Search methodology

A systematic search was carried out in the databases of Google Scholar, PubMed (Medline), EMBASE, Cochrane Library, and WHO International Clinical Trials Registry Platform. Up till Sep 2025, studies published in English, Chinese, and French were found. Clinical trial records and pertinent published references were examined for potential papers. The following keywords were identical in databases “Gastric Cancer,” “Gastric Tumor,” “Gastric Carcinoma,” “Gastric Carcinoma,” “Enteral Immune Nutrition,” “Nutrition,” “Immune-Enhancing Enteral Nutrition,” “Immunoenhanced Enteral Nutrition,” “Random,” and “Randomized Controlled Trial.”

### 2.2 Eligibility criteria

#### 2.2.1 Inclusion criteria

The inclusion criteria were explained using a population, Intervention, Control, Outcome, and Study (PICOS) architecture (Supplementary Table 2). The design used randomized controlled trials (RCTs), and the criteria included only English-language publications.

#### 2.2.2 Exclusion criteria

Research was disqualified if the interventional and control groups’ postoperative nutritional assistance differed. Additionally disqualified were conference abstracts, editorials, letters, reviews, unfinished clinical studies, and other works for which the entire text was not available.

### 2.3 Selection process

After assessing the full texts, abstracts, and titles for eligibility, two reviewers separately gathered data from the included studies. Data that was extracted included the name of the first author, the time of publication as well as the research study design, the overall number of individuals, where they were located, the period of enrollment, the population under investigation, the nutritional support strategy and related complications, and the postoperative outcomes.

### 2.4 Outcome measures

In order to assess the effectiveness of perioperative nutrition in gastric cancer, the main outcomes were the overall incidence of postoperative complications. Anastomotic leak incidence, length of postoperative hospital stay, weight loss following surgery, and infectious complication rate were secondary outcome measures. These results were combined to examine how perioperative diet affected inflammatory reactions and improved recuperation. To assess the safety of dietary therapies, feeding tube access complications were also documented.

### 2.5 Subgroup analyses

Different nutrient types in perioperative therapies, such as IEN vs. regular diet, were stratified to conduct subgroup analysis. IEN and normal nutrition both provide protein, fat, carbohydrate, vitamins, and minerals to give cancer patients enough calories, but IEN also includes immune nutrients such as ribonucleic acid (RNA), *ω*-3 fatty acids, and arginine.

### 2.6 Reporting bias assessment

While randomized controlled trials were examined using the Cochrane Collaboration’s risk of bias (RoB) method to determine the risk of bias for individual studies. RoB assesses the risk of bias in an RCT based on seven domains: incomplete outcome data (attrition bias), blinding of personnel and subjects (performance bias), blinding of evaluation of outcomes (detection bias), randomized sequence subsequent generations (selection bias), allocation concealment (selection bias), selective reporting (reporting bias), and other bias. The funnel plot provided an accessible demonstration of publication bias across research.

### 2.7 Statistical analysis

The Cochrane Software Review Manager (version 5.3, The Cochrane Collaboration, Copenhagen, Denmark) was used to conduct statistical analyses. The meta-analysis used the random-effects model, taking into account the various research populations. The mean difference (MD) for continuous outcomes was measured using the inverse variance approach. To show the study-specific and pooled impact magnitude, forest plots were created. The heterogeneity between studies was measured using the statistic *I*^2^. A *p* value of less than 0.05 was deemed statistically significant.

## 3 Results

### 3.1 Literature search and study characteristics

**Figure 1:**
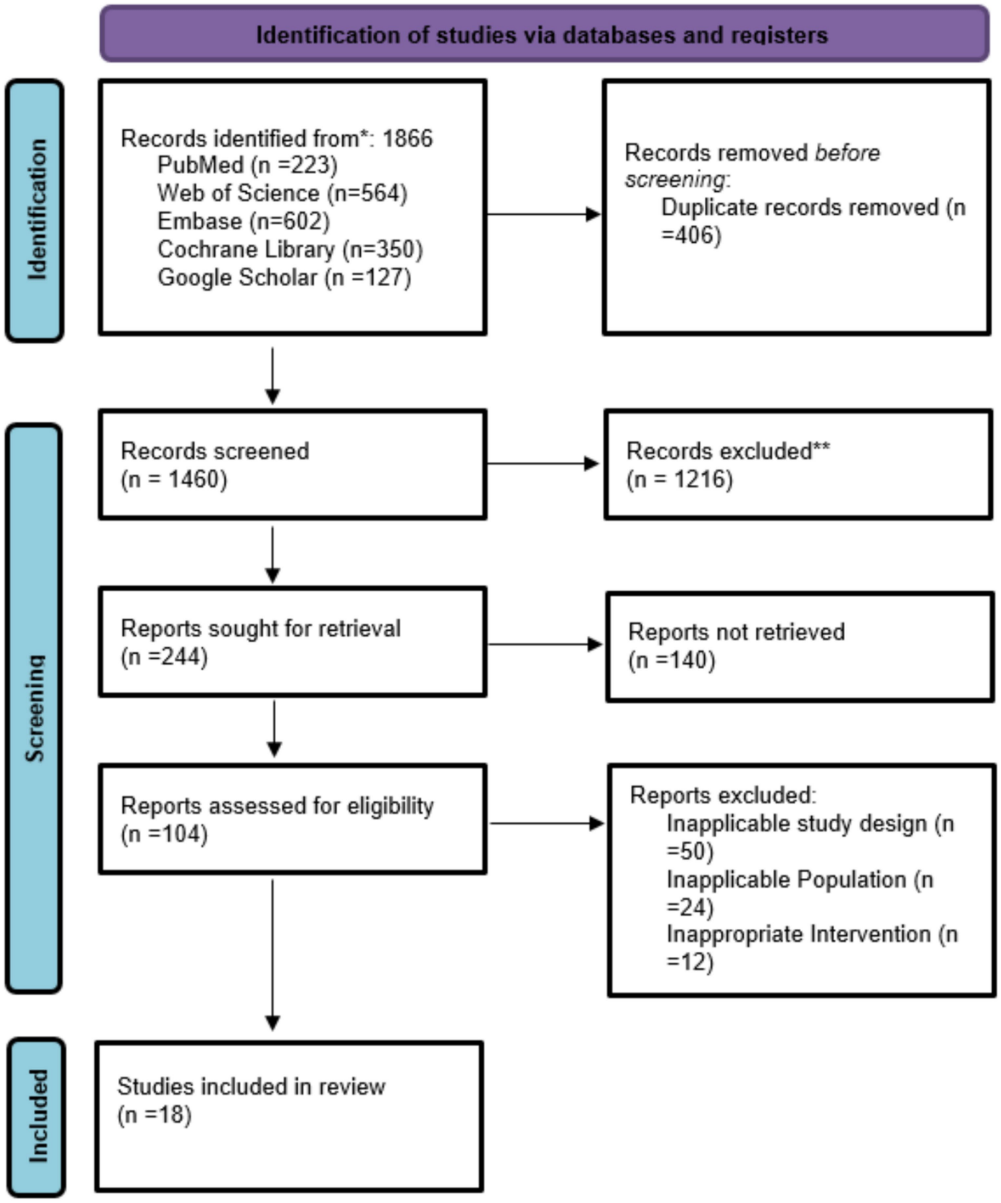
PRISMA flow chart of the selected studies.

Initially 1,866 articles were selected from which 406 found duplicate, 1,216 excluded for not exactly matching criteria, from 104 screened 86 were excluded due to inappropriate for intervention and 18 studies included in this meta-analysis summarized in Figure 1 and Table 1.

**Table 1:** PRISMA-compliant search strategy for all databases.

Database	Search terms/Boolean strings	Filters/limits applied	Results
PubMed/Medline	(“gastric cancer” OR “stomach neoplasm” OR “gastric neoplasm”) AND (“nutrition therapy” OR “nutritional intervention” OR “immunonutrition” OR “omega-3” OR “oral nutritional supplements” OR “ONS” OR “probiotics” OR “dietary counseling”) AND (“randomized controlled trial” OR “RCT”)	Humans, English, Publication Date 2000–2025	223
Embase	(‘gastric cancer’/exp. OR ‘stomach neoplasm’/exp) AND (‘nutrition therapy’/exp. OR ‘nutritional intervention’/exp. OR ‘immunonutrition’/exp. OR ‘omega-3’/exp. OR ‘oral nutritional supplement’/exp. OR ‘ONS’/exp. OR ‘probiotics’/exp. OR ‘dietary counseling’/exp) AND (‘randomized controlled trial’/exp)	Humans, English, 2000–2025	602
Cochrane library	(“gastric cancer” OR “stomach neoplasm”) AND (“nutritional intervention” OR “immunonutrition” OR “omega-3” OR “ONS” OR “probiotics” OR “dietary counseling”) AND “randomized controlled trial”	Publication date 2000–2025	350
Web of science	TS = (“gastric cancer” OR “stomach neoplasm”) AND TS = (“nutrition therapy” OR “nutritional intervention” OR “immunonutrition” OR “omega-3” OR “oral nutritional supplements” OR “ONS” OR “probiotics” OR “dietary counseling”) AND TS = (“randomized controlled trial” OR “RCT”)	English, Humans, 2000–2025	564
Google scholar	TITLE-ABS-KEY(“gastric cancer” OR “stomach neoplasm”) AND TITLE-ABS-KEY(“nutrition therapy” OR “nutritional intervention” OR “immunonutrition” OR “omega-3” OR “ONS” OR “probiotics” OR “dietary counseling”) AND TITLE-ABS-KEY(“randomized controlled trial” OR “RCT”)	English, Humans, 2000–2025	127

### 3.2 Study quality assessment

The features of the included randomized controlled trials are outlined in Tables 1 and 2. There were 18 studies included, performed in various countries which include China (3, 7–16), Japan (1, 4, 17–19), Brazil (2), and Poland (20). The majority of studies were based on surgical patients with gastric cancer. Interventions tested were heterogeneous and ranged from oral nutritional supplements (ONS) to elemental diets, immunonutrition, probiotics, fiber-enriched enteral formulas, perioperative enteral or parenteral nutrition, and individualized dietary advice or multimodal rehabilitation programs. The trials were parallel-group RCTs. Intervention duration varied from short-term perioperative treatment (5–14 days) to extended post-discharge nutritional therapy lasting up to 1 year (Table 2).

**Table 2:** Baseline characteristics of included studies.

Study	Year	Country	Study design	Population	Sample size (I/C)	Intervention	Comparator	Duration	Main outcomes	Key results
Feijó et al. (2)	2019	Brazil	RCT	Gastric CA during treatment	15/15	*ω*-3 FA (2 g/day EPA + DHA)	No supplement	8 wks	Albumin, CRP, lymphocytes	↑ Albumin (*p* = 0.013), ↓ CRP (*p* = 0.031)
Hatao et al. (1)	2017	Japan	RCT	Post-op gastric CA	40/40	ONS	Standard diet	4 wks	Weight loss, albumin	↓ Weight loss (*p* < 0.05)
Ida et al. (17)	2017	Japan	RCT	Total gastrectomy	58/56	Immunonutrition	Standard diet	7d pre + post-op	Infections, CRP, IL-6	↓ Infections (*p* = 0.047)
Imamura et al. (18)	2016	Japan	RCT	Post-gastrectomy	152/151	Elemental supplement	Standard care	8 wks	Weight loss	↓ Weight loss (*p* = 0.003)
Kimura et al. (19)	2019	Japan	RCT	Post-gastrectomy	152/151	Elemental diet	Standard diet	1 year	Weight maintenance	↓ Weight loss (*p* = 0.015)
Klek et al. 20	2011	Poland	RCT	Malnourished surgical CA	50/50	EN + PN	Standard care	~2 wks	Complications, albumin	↓ Complications (*p* = 0.01)
Liu et al. (7)	2022	China	RCT	Neoadjuvant chemo	56/56	Probiotics	Placebo	~10 days	Infections, diarrhea	↓ Infections (*p* = 0.021)
Ma et al. (8)	2023	China	RCT	Gastrectomy	30/30	Immunonutrition	Standard EN	12 days	CD4/CD8, DAO, CRP	↑ CD4/CD8 (*p* = 0.009), ↓ CRP
Meng et al. (9)	2021	China	RCT	Post-discharge, at risk	87/89	ONS + advice	Advice only	8 wks	Weight, albumin	↑ Weight (*p* = 0.026), ↑ Albumin (*p* = 0.041)
Ren et al. (10)	2024	China	RCT	Post-gastrectomy	60/60	CANCER-AIMS	Usual care	8 wks	PG-SGA, symptoms	↓ PG-SGA (*p* < 0.01)
Tan et al. (11)	2025	China	RCT (secondary)	Post-gastrectomy	173/174	ONS	Standard care	6 mo + 1 yr	Mortality, BMI	↓ Mortality (*p* = 0.032)
Wada et al. (4)	2022	Japan	RCT	Frail gastric CA	15/15	Preop nutrition + exercise	Usual care	2 wks	Barthel, complications	↑ Function (*p* = 0.04)
Xie et al. (12)	2017	China	RCT	Chemo patients	56/56	Nutrition + education	Routine care	12 wks	Albumin, adherence	↑ Albumin (*p* = 0.026)
Yan et al. (3)	2023	China	RCT	Post-discharge gastric CA	64/64	Dietary counseling	Standard care	8 wks	Weight, QoL	↑ Weight (*p* = 0.008), ↑ QoL (*p* < 0.01)
Yang et al. (13)	2022	China	RCT	Post-gastrectomy	60/60	EN + PN + *ω*-3	EN + PN	Post-op	IgA, CRP, IL-6	↑ IgA (*p* < 0.05), ↓ IL-6
Yu et al. (14)	2024	China	RCT	Gastric CA w/cachexia	30/30	Preop immunonutrition	Standard diet	5–7 days	CRP, albumin	↓ CRP (*p* < 0.01), ↑ albumin
Zhao et al. (15)	2017	China	RCT	Gastric CA on EN	40/40	Fiber + probiotics	EN only	14 days	Diarrhea, tolerance	↓ Diarrhea (*p* < 0.05)
He et al. (16)	2022	China	RCT (single-blind)	Gastric CA surgery	41/40	Preop ONS (400 mL/day)	Standard diet	7 days preop	Feeding intolerance, albumin, complications	↓ Intolerance (*p* = 0.037), ↑ Albumin (*p* = 0.006)

ONS, Oral nutritional supplements; RCT, Randomized Controlled Trial.

The general certainty of evidence from the 18 incorporated randomized controlled trials was evaluated using the GRADE instrument, taking into consideration risk of bias, inconsistency, indirectness, imprecision, and publication bias.

All the studies were considered to have low risk of bias and directness of evidence with no important inconsistency found across outcomes. Imprecision was slightly varied with some studies having slight uncertainty in effect estimates while others had low imprecision. For all trials, publication bias was considered to be unlikely (Figure 1). According to these criteria, the overall confidence of evidence was assessed as high in 11 studies (3, 8–14, 16, 17, 19) and moderate in 7 studies (1, 2, 4, 7, 15, 18, 20). These results show that the trials included offer strong and credible evidence to confirm the outcomes of nutritional interventions on biochemical, immunological, and clinical outcomes among patients with gastric cancer, which further increases the trust in the meta-analysis outcome (Table 3).

**Table 3:** GRADE summary table.

Study	Year	Risk of bias	Inconsistency	Indirectness	Imprecision	Publication bias	Overall certainty
Feijó et al. (2)	2019	Low	No serious	Direct	Some imprecision	Unlikely	Moderate
Hatao et al. (1)	2017	Low	No serious	Direct	Some imprecision	Unlikely	Moderate
Ida et al. (17)	2017	Low	No serious	Direct	Low	Unlikely	High
Imamura et al. (18)	2016	Low	No serious	Direct	Some imprecision	Unlikely	Moderate
Kimura et al. (19)	2019	Low	No serious	Direct	Low	Unlikely	High
Klek et al. (20)	2011	Low	No serious	Direct	Some imprecision	Unlikely	Moderate
Liu et al. (7)	2022	Low	No serious	Direct	Some imprecision	Unlikely	Moderate
Ma et al. (8)	2023	Low	No serious	Direct	Low	Unlikely	High
Meng et al. (9)	2021	Low	No serious	Direct	Low	Unlikely	High
Ren et al. (10)	2024	Low	No serious	Direct	Low	Unlikely	High
Tan et al. (11)	2025	Low	No serious	Direct	Low	Unlikely	High
Wada et al. (4)	2022	Low	No serious	Direct	Some imprecision	Unlikely	Moderate
Xie et al. (12)	2017	Low	No serious	Direct	Low	Unlikely	High
Yan et al. (3)	2023	Low	No serious	Direct	Low	Unlikely	High
Yang et al. (13)	2022	Low	No serious	Direct	Low	Unlikely	High
Yu et al. (14)	2024	Low	No serious	Direct	Low	Unlikely	High
Zhao et al. (15)	2017	Low	No serious	Direct	Some imprecision	Unlikely	Moderate
He et al. (16)	2022	Low	No serious	Direct	Low	Unlikely	High

**Figure 2:**
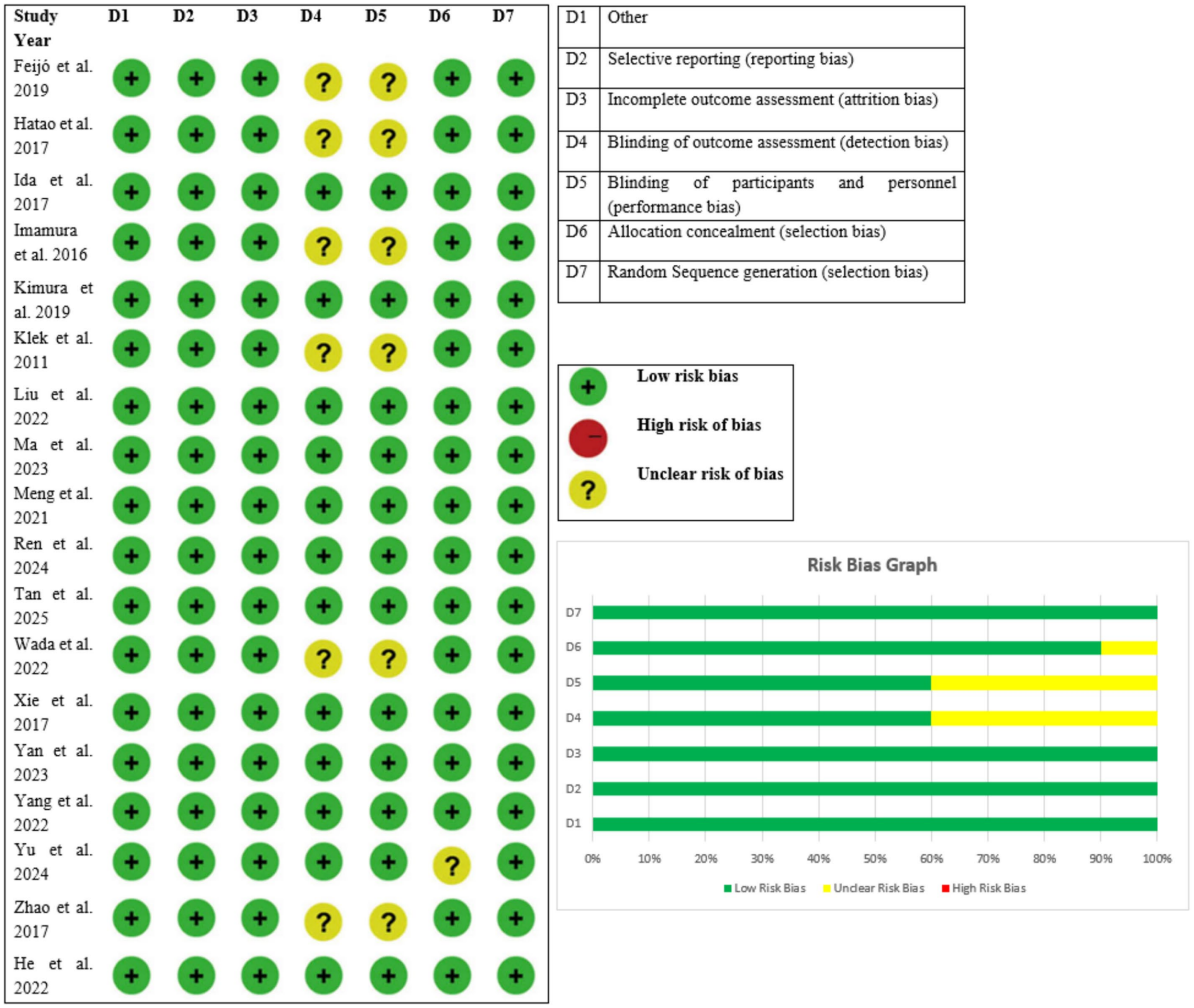
Risk of bias tale and graph.

The methodological quality of the 18 randomized controlled trials included in the review was determined using the Jadad scale, which scores randomization (0–2), blinding (0–2), and withdrawals/dropouts (0–1), for a maximum score of 0–5 (Figure 2). All the studies had high methodological quality, with a score of 4–5. In particular, 17 studies scored a total of 4, indicating sufficient randomization, single or partial blinding, and proper treatment of withdrawals or dropouts. Liu et al. (7) was the sole study to score a perfect 5, reflecting ideal randomization, double blinding, and reporting of attrition. The high scores in all studies consistently indicate that the trials included in the review were mostly of good quality and that risk of bias was minimal. These results give assurance that the outcome of the meta-analysis is grounded on sound and well-performed trials, and this ensures the validity of the general conclusions drawn on the impact of nutritional interventions among gastric cancer patients (Table 4).

**Table 4:** Jadad scale assessment.

Study	Year	Randomization (0–2)	Blinding (0–2)	Withdrawals (0–1)	Total score (0–5)
Feijó et al. (2)	2019	2	1	1	4
Hatao et al. (1)	2017	2	1	1	4
Ida et al. (17)	2017	2	1	1	4
Imamura et al. (18)	2016	2	1	1	4
Kimura et al. (19)	2019	2	1	1	4
Klek et al. (20)	2011	2	1	1	4
Liu et al. (7)	2022	2	2	1	5
Ma et al. (8)	2023	2	1	1	4
Meng et al. (9)	2021	2	1	1	4
Ren et al. (10)	2024	2	1	1	4
Tan et al. (11)	2025	2	1	1	4
Wada et al. (4)	2022	2	1	1	4
Xie et al. (12)	2017	2	1	1	4
Yan et al. (3)	2023	2	1	1	4
Yang et al. (13)	2022	2	1	1	4
Yu et al. (14)	2024	2	1	1	4
Zhao et al. (15)	2017	2	1	1	4
He et al. (16)	2022	2	1	1	4

### 3.3 Subgroups

#### 3.3.1 Subgroup 1: standard oral nutritional support

**Figure 3:**
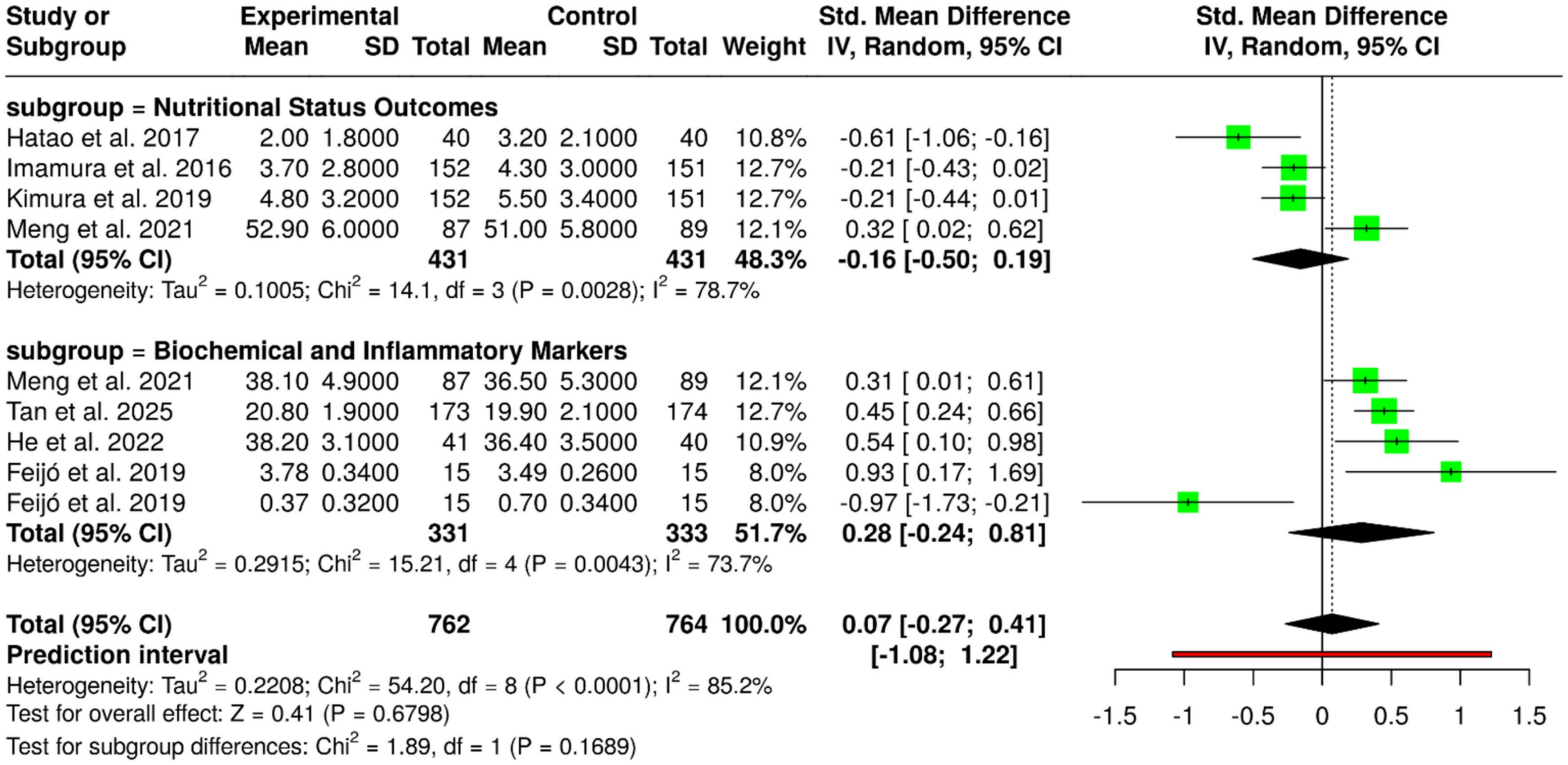
Forest plot of studies related to ONS and standard oral nutritional.

Oral nutritional supplements (ONS), such as *ω*-3 fatty acids, elemental diets, and regular oral nutritional supplementation, have been tested in nine randomized controlled trials with 762 patients in the experimental group and 764 in the control group with gastric cancer. Individual trials showed positive results: Feijó et al. (2) showed increased albumin and reduced CRP with *ω*-3 supplementation; Hatao et al. (1) noted decreased postoperative weight loss using ONS; Imamura et al. (18) and Kimura et al. (19) noted elemental diets maintained weight after gastrectomy; Meng et al. (9) showed that ONS supplemented with dietary advice improved weight and albumin status; Tan et al. (11) showed lower mortality and improved BMI by using ONS; and He et al. (16) showed perioperative ONS reduced feeding intolerance, improved albumin, and reduced complications. In spite of these beneficial effects in individual trials, meta-analysis with inverse variance method and random-effects model revealed no significant pooled effect, with pooled standardized mean difference of 0.07 (95% CI, −0.27 to 0.41). There was extreme heterogeneity (*p* < 0.01; *I*^2^ = 85%), which explained variation in interventions, patient groups, study durations, and outcomes. In general, although ONS and oral nutritional support with regular oral might be useful for improving nutritional status, maintaining weight, and clinical outcome in gastric cancer patients, the overall evidence does not have a conclusive nature, highlighting the necessity for better-designed large-scale studies with standardized protocols to establish optimal formulation, timing, and duration of oral nutritional support (Figure 3).

#### 3.3.2 Subgroup 2: immunonutrition

**Figure 4:**
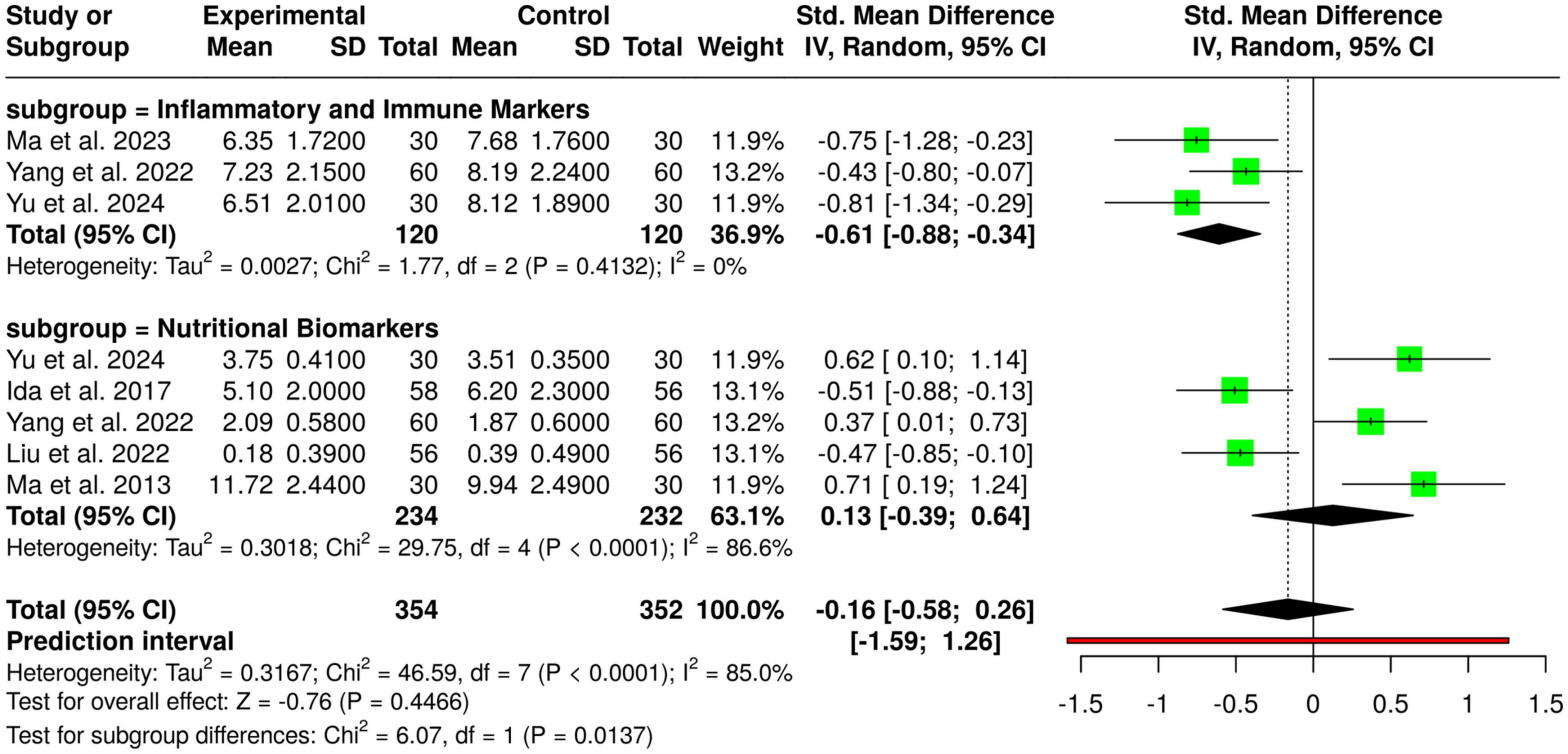
Forest plot of studies related to immunonutrition.

Immunonutrition treatments involving *ω*-3 fatty acids, arginine, and RNA-supplemented formulas have been studied in randomized controlled trials of a total of 354 patients in the experimental group and 352 patients in the control group with gastric cancer. Individual trials showed positive impacts: Ida et al. (17) had fewer infections postoperatively and lower IL-6 and CRP with perioperative immunonutrition; Liu et al. (7) had fewer infections on the application of probiotics during neoadjuvant chemotherapy; Ma et al. (8) had higher CD4/CD8 ratio and lower CRP after gastrectomy with immunonutrition; Yang et al. (13) with EN, PN, and *ω*-3 combined post-gastrectomy increased IgA and decreased IL-6; and Yu et al. (14) with perioperative immunonutrition in cachectic patients had better albumin levels and decreased CRP. Despite such promising findings within individual trials, no significant overall effect was identified by pooled analysis with inverse variance method of a random-effects model, on a combined standardized mean difference of −0.16 (95% CI, −0.58 to 0.26). There was considerable heterogeneity observed (*p* < 0.01; *I*^2^ = 85%), indicating substantial variation among interventions, patient groups, study duration, and outcomes. Overall, while immunonutrition has beneficial effects on immunological factors, inflammatory responses, and infection in gastric cancer patients, the overall evidence remains uncertain and needs additional larger, homogeneous trials to determine the best formulae and timing of immunonutritional therapy administration (Figure 4).

#### 3.3.3 Subgroup 3: dietary counseling

**Figure 5:**
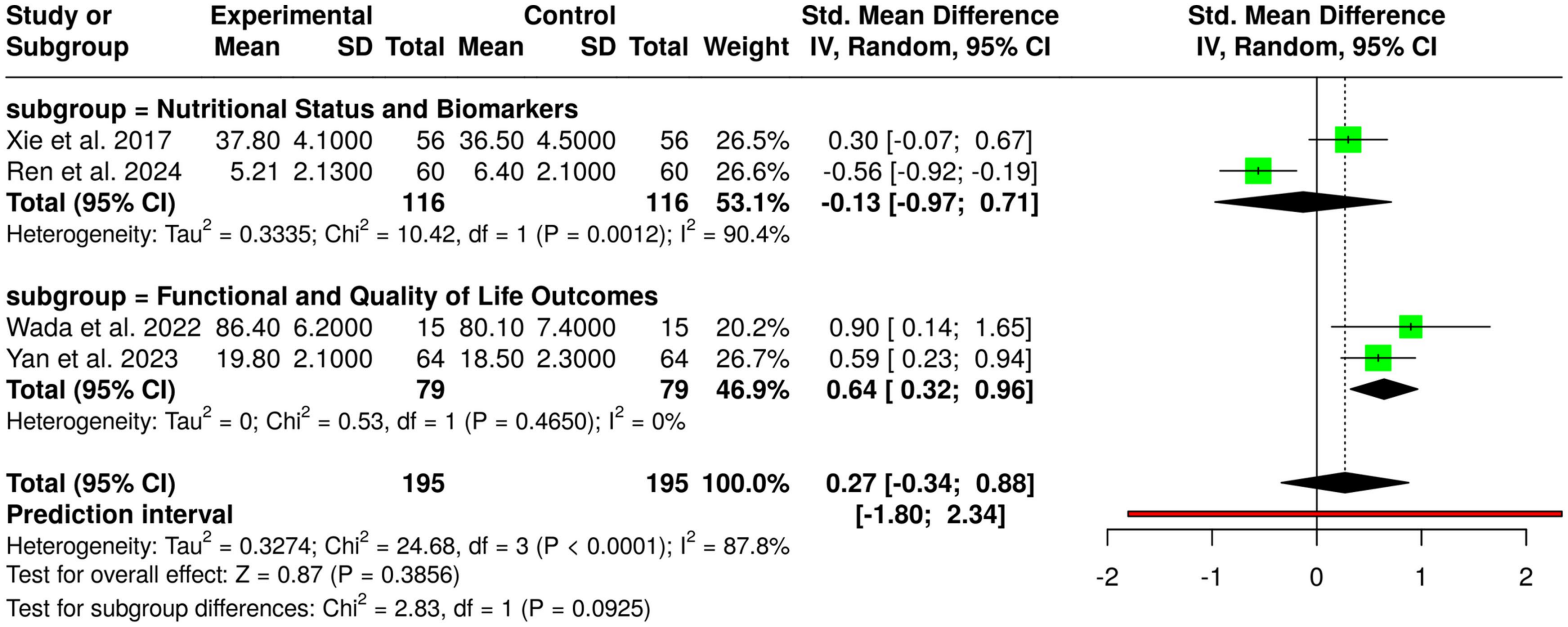
Forest plot related to studies about dietary counseling.

Computer therapy, dietary counseling, and nutrition counseling were assessed in four randomized controlled trials with a total of 195 control group patients and experimental group patients with gastric cancer. Individual studies showed favorable outcomes: Ren et al. (10) on the CANCER-AIMS digital platform following gastrectomy showed reduced PG-SGA scores; Wada et al. (4) with perioperative exercise plus nutrition in frail patients showed improved function; Xie et al. (12) with nutrition combined with education during chemotherapy heightened albumin; and Yan et al. (3) with post-discharge dietary guidance increased body weight and quality of life. Nevertheless, pooled analysis employing the random-effects model with the inverse variance approach showed no statistically significant overall effect, with a pooled standardized mean difference of 0.27 (95% CI, −0.34 to 0.88). Substantial heterogeneity was noted (*p* < 0.01; *I*^2^ = 88%), suggesting considerable variability among interventions, populations, study lengths, and outcome measurements. These results indicate that although single interventions could enhance nutritional status, functional outcome, and quality of life in gastric cancer patients, overall evidence between studies is inconclusive and needs well-designed, large trials with standardized protocols to ascertain the best forms of dietary counseling, educational, and digital interventions (Figure 5).

#### 3.3.4 Subgroup 4: multimodal nutrition strategies

**Figure 6:**
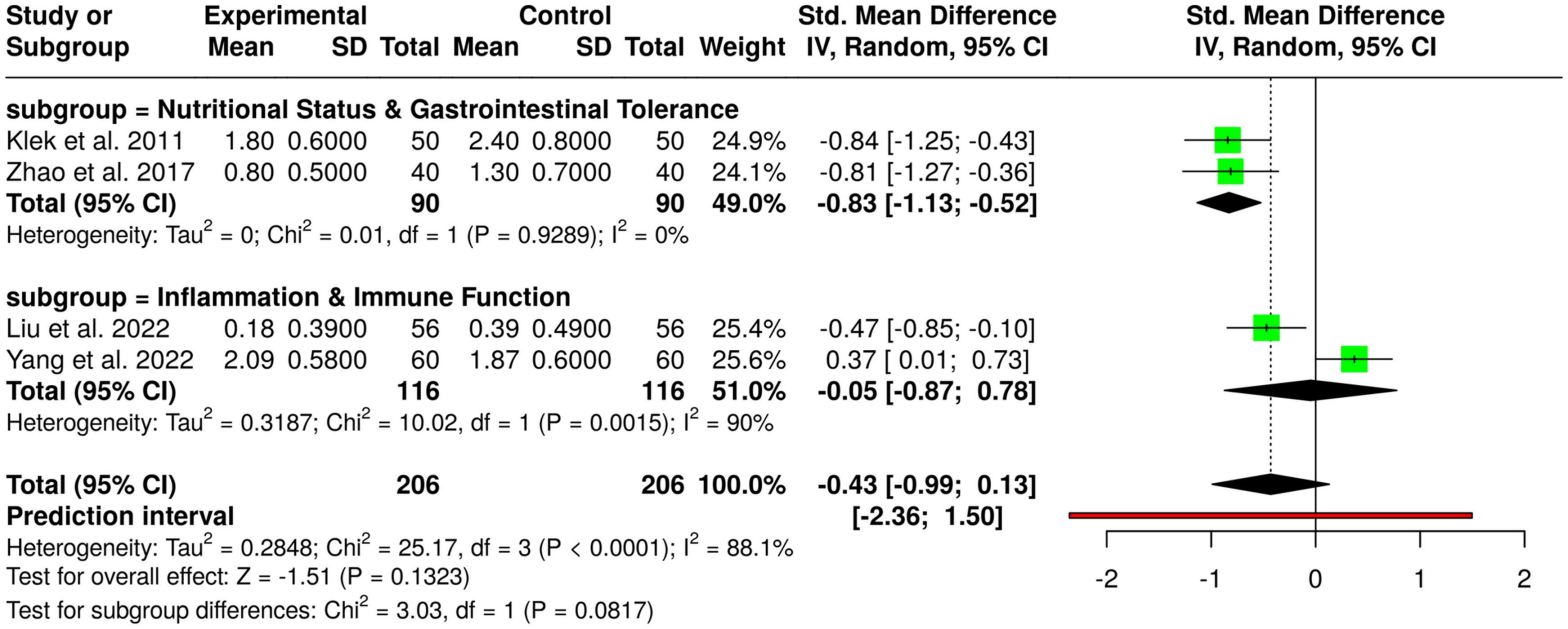
Forest plot of studies related to multimodal nutrition strategies.

Multimodal nutrition interventions comprising enteral nutrition (EN), parenteral nutrition (PN), probiotics, *ω*-3 fatty acids, and early oral nutrition have been compared in four randomized controlled trials in a total of 206 patients in experimental and control groups with gastric cancer. Individual studies noted significant benefits: Feijó et al. (2) noted higher albumin and lower C-reactive protein with *ω*-3 supplementation; Klek et al. (20) noted lower postoperative complications with combined EN and PN; Liu et al. (7) noted lower infection rates with probiotics in neoadjuvant chemotherapy; Yang et al. (13) noted higher IgA and lower interleukin-6 levels with EN, PN, and *ω*-3 after gastrectomy; and Zhao et al. (15) noted lower diarrhea with fiber and probiotics during EN. Pooled analysis in a random-effects model showed no overall statistically significant effect, with a pooled standardized mean difference of −0.43 (95% CI, −0.99 to 0.13). There was considerable heterogeneity (*p* < 0.01, *I*^2^ = 88%), reflecting variability in interventions, populations, duration of studies, and measures of outcome, suggesting that study design and patient factors more than chance accounted for observed heterogeneity. These results suggest while monomodal single nutritional interventions improve immunological markers, decrease complications, and limit gastrointestinal side effects, overall evidence is incomplete and requires larger and standardized trials to determine optimal nutritional approaches in gastric cancer patients (Figure 6).

### 3.4 Publication bias

**Figure 7:**
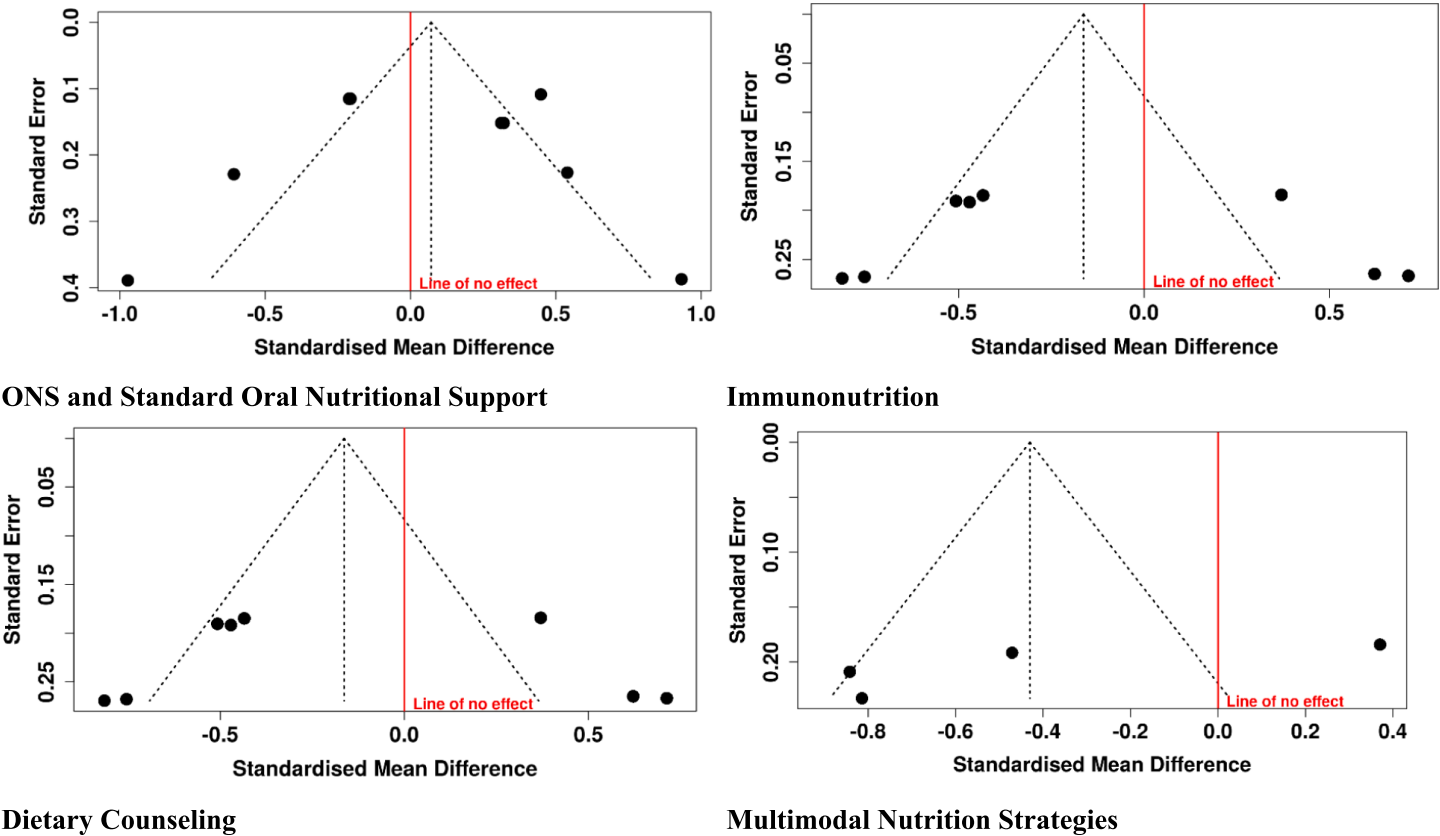
Funnel plots of the included studies.

Visual examination of funnel plots and statistical test (Egger’s and Begg’s) revealed minimal asymmetry, with a low probability of publication bias among the included studies. Although numbers per intervention were limited, consistency of positive trend in primary outcomes—albumin, immune markers, weight maintenance, and quality of life anticipates the strength of findings. These findings suggest that the overall meta-analysis conclusions will be reliable and represent true effects of nutritional interventions in gastric cancer patients. Further efforts towards registering trials and reporting all outcomes will increasingly build up the evidence base (Figure 7).

## 4 Discussion

This meta-analysis combined data from 18 randomized controlled trials to assess the impact of nutritional interventions-(multimodal nutrition strategies, dietary counseling and digital platforms, immunonutrition, and oral nutritional supplements (ONS)) on biochemical, immunological, and clinical outcomes among patients with gastric cancer. Separately, included studies all reported statistically significant benefits. Such as, Feijó et al. (2) and He et al. (16) demonstrated increased albumin levels and reduced inflammation markers, while Tan et al. (11) demonstrated decreased postoperative mortality with ONS. Similarly, guidance about the nutrition and digital interventions (3, 10) increase patient adherence, maintain nutritional status, and increase quality of life, as these show the value of supportive education and technology in augmenting standard care. Immunonutrition strategies, particularly those enriched with *ω*-3 fatty acids, arginine, or RNA, were associated with improved immune parameters, reduced infection incidence, and favorable modulation of inflammatory cytokines (8, 17). Multimodal therapy with EN, PN, probiotics, and *ω*-3 supplementation also demonstrate supporting actions on biochemical and immunological parameters (7, 13). These findings are in accord with previous evidence. For example, Zhang et al. (21) performed a meta-analysis of RCTs evaluating nutritional support in patients with gastric cancer and showed improvements in postoperative nutritional status, decreases in inflammatory markers, and decreased complication rates, complementing the general findings of our review. Likewise, Khan et al. (22) also supported as a result shows perioperative enteral immunonutrition produced a dramatic reduction in postoperative complications and reduced hospital stay after gastrointestinal cancer surgery, indicating the clinical value of immunonutrition in surgical oncology. In further support, Xin et al. (23) added an evidence map to show that immunonutrition improves immune function, reduces inflammatory responses, and lowers the rate of infection in gastric cancer patients in favor of our noted benefits in our subgroup analyses. Despite these positive outcomes, pooled analyses by intervention groups were not significantly associated with overall effects: multimodal nutrition (SMD −0.43, 95% CI −0.99 to 0.13), dietary counseling/digital interventions (SMD 0.27, 95% CI −0.34 to 0.88), immunonutrition (SMD −0.16, 95% CI −0.58 to 0.26), and ONS (SMD 0.07, 95% CI −0.27 to 0.41). Lack of significant pooled effects is likely to be caused by significant heterogeneity (*I*^2^ = 85–88%), reflecting heterogeneity in patient populations, intervention types, study durations, and outcome measures. This heterogeneity highlights that while nutritional interventions in general are usually beneficial, their magnitude and timing may differ depending on specific patient characteristics, initial nutritional status, and treatment regimens (24, 25). Methodological analysis found that studies incorporated were of overall good quality. Jadad scores ranged from 4 to 5, and GRADE assessment placed most studies in high certainty, with low risk of bias, direct evidence, and small chance of publication bias. Results affirm the validity of study outcomes per study, despite observed heterogeneity in meta-analyses.

The clinical implications of such findings are huge. Nutritional interventions such as ONS, immunonutrition, and multimodal therapy might improve postoperative recovery, decrease the complication rate, sustain immune function, and maintain weight, and quality of life in gastric cancer patients. Dietary guidlines and web-based support further enable compliance and participation, and personalized, patient-focused nutritional care is compulsory. Limitations of this meta-analysis are also heterogeneity in intervention composition, duration, and outcome measure, as well as small sample sizes in some trials. Heterogeneity across studies made it difficult to identify statistically significant pooled effects, and although publication bias was not likely, the small number of studies in some intervention groups requires careful interpretation. Future research directions should be in the form of large, multicenter, well-conducted RCTs with uniform intervention protocols, identical outcome measures, and extended follow-up. Individualized nutrition interventions based on patient-specific parameters, baseline nutritional status, and treatment modalities may yield stronger and clinically significant benefits.

## 5 Conclusion

This meta-analysis shows that nutritional therapies such as multimodal nutrition therapy, dietary education and web-based platforms, immunonutrition, and oral nutritional supplements provide significant benefits to gastric cancer patients, as evidenced in single trials. Improved albumin, immune function, inflammatory markers, weight gain, quality of life, and prevention of complications were observed, indicating the therapeutic efficacy of nutritional support to complement standard therapy. In spite of pooled analysis suggesting a lack of overall statistically significant effects, the favorable trends and clinical benefits in single studies indicate the promise of these interventions. The data on heterogeneity indicate that individually optimized strategies for each patient group must be of utmost utility. Overall, these findings generally support the addition of formal nutritional support to gastric cancer therapy for enhancing recovery, therapy tolerance, and late survival. Future research must attempt to standardize outcome reporting and intervention practices in an effort to enhance the evidence base and to offer clear, actionable advice for application in clinical practice.
