# Correction: Perioperative nutritional interventions for gastric cancer patients: a meta-analysis of surgical outcomes

**DOI:** 10.3389/fnut.2025.1745856

**Published:** 2025-11-25

**Authors:** QiJun Li, Chen Chen, XiaoHong Wang

**Affiliations:** General Surgery (Gastroenterology), Huadong Hospital, Fudan University, Shanghai, China

**Keywords:** gastric cancer, nutritional interventions, immunonutrition, oral nutritional supplements, dietary counseling, multimodal nutrition, randomized controlled trials, meta-analysis

In the published article, the name of the author, QiJun Li was erroneously spelled “JunQi Li”. In the published article, the name of the author XiaoHong Wang was erroneously spelled “HongXiao Wang”.

In the published article, the **Author contributions** statement was erroneously given as [JL: Methodology, Project administration, Writing – original draft, Writing – review & editing. CC: Methodology, Writing – review & editing. HW: Supervision, Writing – original draft]. The correct **Author contributions** statement is [QL: Methodology, Project administration, Writing – original draft, Writing – review & editing. CC: Methodology, Writing – review & editing. XW: Supervision, Writing – original draft].

The original version of this article has been updated.

